# Influence of angle alpha on visual quality after implantation of extended depth of focus intraocular lenses

**DOI:** 10.1186/s12886-022-02302-4

**Published:** 2022-02-17

**Authors:** Miaomiao Qin, Min Ji, Tianqiu Zhou, Yurong Yuan, Jiawei Luo, Pengfei Li, Ying Wang, Xiaojuan Chen, Wei Chen, Huaijin Guan

**Affiliations:** Eye Institute, Affiliated Hospital of Nantong University, 20 Xisi Road, Nantong, Jiangsu China

**Keywords:** Angle alpha, Visual quality, Extended depth of focus intraocular lenses

## Abstract

**Background:**

To assess postoperative changes in angle alpha, and to evaluate the postoperative visual quality of patients with different angle alpha values after implantation of extended depth of focus (EDOF) intraocular lenses (IOLs).

**Methods:**

Seventy-nine eyes of 79 patients who had phacoemulsification with EDOF IOLs implantation were enrolled. A cut-off value of 0.3 mm, 0.4 mm, and 0.5 mm in preoperative angle alpha was chosen to divide eyes into groups. Distance, intermediate, and near visual acuities, modulation transfer function (MTF), and aberrations were recorded during a 6-month follow-up. A patient questionnaire was completed.

**Results:**

There were no significant differences in angle alpha postoperatively compared to preoperatively. No significant differences were found in visual acuity and MTF between all groups. With 5 mm pupil diameter, there were significant differences of higher-order aberrations and spherical aberration in ocular aberration and internal aberration between angle alpha<0.4 mm and angle alpha≥0.4 mm. Additionally, significant differences of coma were also added in cut-off value of 0.5 mm. When the value of angle alpha is 0.4 mm or higher, there were significant differences in the score of halos and glare.

**Conclusions:**

Angle alpha did not affect visual acuity, but the value of 0.4 mm or higher in angle alpha affected the visual quality under scotopic conditions and occurrence of halos and glare. For patients with 0.4 mm or higher in angle alpha, the choice to implant a EDOF IOL should be carefully considered.

**Supplementary Information:**

The online version contains supplementary material available at 10.1186/s12886-022-02302-4.

## Background

With the improvement in people’s living standards and technology of intraocular lenses (IOLs), cataract surgery has been transformed from simple blindness prevention to refractive cataract surgery. The ultimate goal for both surgeons and cataract patients is not only to achieve better visual acuity at far, intermediate, and near distance, but also to improve satisfactory postoperative outcomes. Therefore, at present, refractive, diffractive, and diffractive-refractive combined multifocal intraocular lenses (MIOLs) are designed to reduce or eliminate the need of wearing spectacles or contact lenses after cataract surgery.

However, some patients have complained the disturbing photic phenomena, including halos and glare, after the implantation of MIOLs, thus affecting their quality of life [1–3]. The human eye is not a perfect optical system, there are more or less deviation between the visual axis and the pupillary axis [4, 5], and between the visual axis and the optical axis in the phakic and pseudophakic eye [6]. These deviations can cause higher-order aberrations (HOAs) postoperatively, thus lead to decreased visual quality [7]. In recent years, more studies have shown the influence of angle kappa on visual quality [1, 2, 8, 9]. Interestingly, previous researches have indicated that angle kappa significantly decreased after phacoemulsification [9, 10], which indicated that angle kappa was a relatively unstable factor for the implantation of MIOLs.

Moreover, so far, some studies indicated that angle alpha could be used as a predictor to achieve postoperative patient satisfaction with MIOLs [11, 12]. Therefore, the aim of this study was to assess postoperative changes in angle alpha, and to analyze and compare the influence of magnitude of angle alpha on visual quality after implantation of extended depth of focus (EDOF) IOLs.

## Methods

The prospective study enrolled patients who scheduled for phacoemulsification surgery with implantation of Tecnis EDOF IOLs (ZXR00; Abbott Medical Optics, Santa Ana, CA) between Mar 2019 and Jan 2020 at the Ophthalmology Center, Affiliated Hospital of Nantong University, Jiangsu, China. The patients were divided into groups based on the magnitude of preoperative angle alpha (chord length alpha = r): Group A1: r<0.3 mm, Group B1: r ≥ 0.3 mm; Group A2: r<0.4 mm, Group B2: r ≥ 0.4 mm; Group A3: r<0.5 mm, Group B3: r ≥ 0.5 mm. It is accepted that patients with angle kappa of less than 0.3 mm had a very low risk for IOL decentration and dissatisfaction [9]. Patients with angle kappa more than 0.4 mm had a high risk of halos and glare after the implantation of diffractive MIOLs [13]. Patients with angle kappa of more than 0.5 mm decreased visual quality with a trifocal IOL [2]. In addition, angle alpha, which was similar to angle kappa, played an important role in the implantation of MIOLs [1]. Therefore, angle alpha sizes of 0.3 mm, 0.4 mm, and 0.5 mm were chosen as the cutoff values. The study was approved by the ethics committee of the Affiliated Hospital of Nantong University and complied with the tenets of the Declaration of Helsinki. Written informed consent was obtained from all patients.

Inclusion criteria included age between 40 and 79 years, corneal astigmatism smaller than 1.00 diopter (D), age-related cataract patients, and lens nuclear hardness grade of 2 to 3. Exclusion criteria were any corneal opacities, poor fixation, strabismus, dry eye, a history of ocular surgery for refractive error and trauma, use of contact lenses, intraoperative or postoperative complications (residual lens fragments, posterior capsule opacification, and so on), and other ocular pathology or neurological lesions that might affect vision.

### Intraocular Lens

The Tecnis EDOF IOL is a posterior chamber IOL made from ultraviolet-filtering hydrophobic acrylic. It has an overall diameter of 13 mm and an optic zone of 6 mm. This IOL is designed to be biconvex. Anterior aspheric surface (spherical aberration-0.27 μm) is designed to correct for the spherical aberration of an average eye [14]. The posterior optic surface is designed an achromatic diffractive surface to correct chromatic aberrations for improvement of contrast sensitivity. The IOL aims to harness a unique pattern of light diffraction that elongates the focus of the eye, improving intermediate vision without compromising quantitative and qualitative vision. The EDOF IOL is available in powers from + 5.00 to + 34.00 D, in 0.50 D increments.

### Surgical technique

All operations were performed by the same experienced surgeon (H-j.G.). A 2.4 mm clear corneal incision (at the steep axis of the cornea) and a 1.0 mm auxiliary incision (at approximately 115° far from clear corneal incision) was created under the guidance of Verion (Alcon, America), and a 5.5 mm continuous curvilinear capsulorhexis was made under the guidance of Verion. Next, hydrodissection, hydrodelineation, and nuclear emulsification were performed. The all chosen EDOF IOLs were then implanted into the capsular bag. All surgeries were successful and there were no intraoperative and postoperative complications.

### Angle alpha measurements

**Fig. 1:**
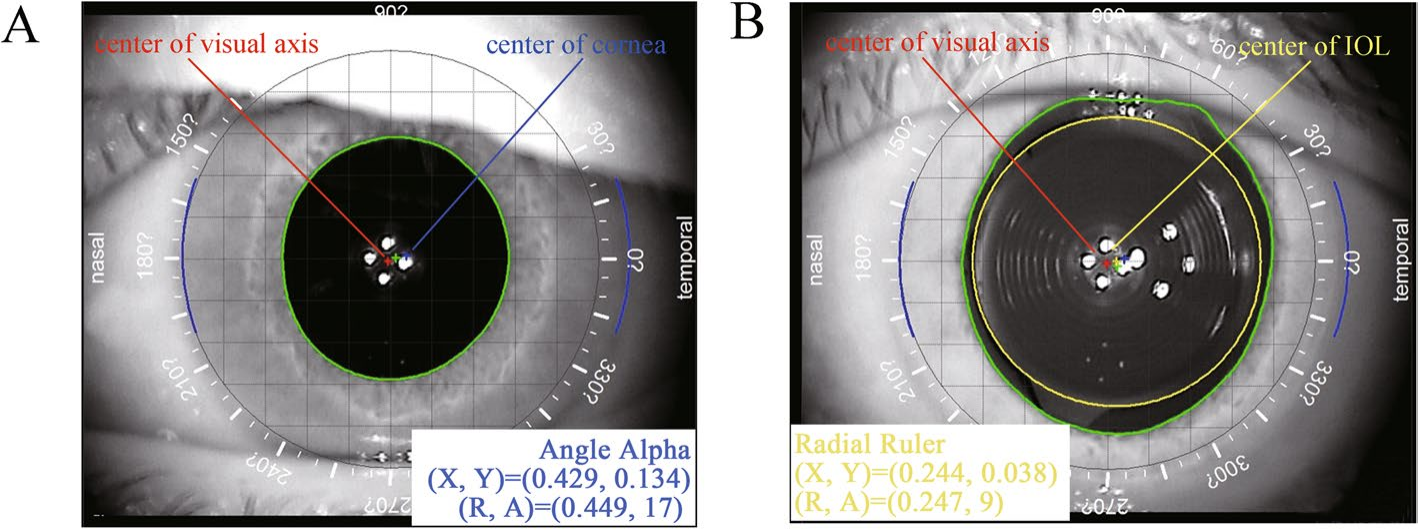
The measurement of the angle alpha and the intraocular lens (IOL) decentration by iTrace aberrometer analyzer. **A** The angle alpha was defined as the distance between the center of the visual axis (red cross) and the center of cornea (blue cross). **B** The IOL decentration was defined as the distance between the center of the visual axis (red cross) and the center of the IOL (yellow cross)

The iTrace aberrometer analyzer (iTrace; Tracey Technologies, Houston, USA) was used by a single experienced examiner (M-m.Q.) to calculate angle alpha within 2 days before surgery and 6 months after surgery in a dark room. Prior to taking examinations, all subjects were asked to blink with the purpose of an optically smooth tear filming over the cornea. The patients placed their chins on a chin rest, and forehead against a forehead strap and fixated on the red light according to the manufacturer’s instructions. An iris image with an infrared camera was captured automatically by the aberrometer to display the center of visual axis and the center of cornea. Angle alpha is the distance in the corneal plane between the center of visual axis and the center of cornea and was reported in polar co-ordinates (distance in millimetres, angle in degrees) (Fig. 1A). To reduce operator-dependent factors, each patient was measured 4 times in consecution and the automatic release mode was used. Only the scans with the minimum of reject points were chosen for statistical analysis.

### Preoperative and postoperative examinations

All patients underwent comprehensive preoperative and postoperative ophthalmologic examinations, which were performed by the same experienced examiner (M-m.Q.). Preoperative examinations were performed within 2 days before surgery, and postoperative examinations were performed at 1 day, 1 week, 1 month, 3 months, and 6 months after surgery. Results were obtained at the 6 months postoperative follow-up, as this is the time for patients to have adapted to implanted EDOF IOLs.

Preoperative examinations included uncorrected distance visual acuity (UDVA), corrected distance visual acuity (CDVA), corneal astigmatism, intraocular pressure, slit-lamp anterior segment evaluation, and fundus examination with the pupil dilated. Biometric IOL power calculations were performed with the ocular biometry Lenstar (LS900; Haag-Streit, Koniz, Switzerland) using the Barrett formula.

Postoperative examinations included UDVA, CDVA, uncorrected intermediate visual acuities (UIVA), corrected intermediate visual acuities (CIVA), uncorrected near visual acuities (UNVA), corrected near visual acuities (CNVA), manifest refraction (sphere, cylinder and manifest refraction spherical equivalent (MRSE)), corneal astigmatism, IOL decentration, modulation transfer function (MTF), ocular aberration, internal aberration, and corneal aberration, and slit-lamp anterior segment evaluation. Distance (5 m), intermediate (80 cm), and near (40 cm) visual acuities recorded in logarithm of the minimum angle of resolution (logMAR) units were measured under the same environment by Snellen visual charts. The IOL decentration was evaluated using iTrace aberrometer analyzer, the instrument identifies the center of the visual axis and the edge of the EDOF IOL when the pupil must be fully dilated (Fig. 1B). MTF, ocular aberration, internal aberration, and corneal aberration were obtained by iTrace aberrometer analyzer with 3 mm and 5 mm pupil diameter under a dark room. In addition, patients were asked to complete a questionnaire survey, the content of the questionnaire survey included the perception of halos and glare from 1 to 4 (1, severe; 2, moderate; 3, slight; and 4, none).

### Statistical analysis

Statistical analysis was done using SPSS software (version 23, SPSS Inc). Visual acuity data were converted into logMAR units for statistical analysis. Values were presented as means ± standard deviations. The normality of data distribution was evaluated using the Kolmogorov–Smirnov test. Depending on the data distribution, Pearson or Spearman rank-order correlation coefficient values were calculated the correlation between angle alpha and the parameters of visual quality. Regression analysis was performed to assess the correlation between angle alpha and the parameters of visual quality. The paired *t*-test was assessed the statistical significance of the difference between postoperative and preoperative angle alpha. The independent sample *t*-test and the Mann–Whitney test was used to compare the differences in visual acuity, MTF, and aberrations in different groups. A *P* value less than 0.05 was considered statistically significant.

## Results

A total of 84 eyes from 84 patients performed phacoemulsification with implantation of EDOF IOLs. Five patients did not return for the follow-up examination 6 months after surgery. At the end of this study, 79 eyes from 79 patients (41 right eyes and 38 left eyes) were comprised in the study. According to the cut-off value in preoperative angle alpha, all eyes were divided into Group A1 (*r*<0.3 mm, 27 eyes) and Group B1 (*r* ≥ 0.3 mm, 52 eyes); Group A2 (*r*<0.4 mm, 41 eyes) and Group B2 (*r* ≥ 0.4 mm, 38 eyes); Group A3 (*r*<0.5 mm, 60 eyes) and Group B3 (*r* ≥ 0.5 mm, 19 eyes). Table 1 shows the preoperative patient parameters. There were no statistically significant differences in these parameters between Group A1 and Group B1, between Group A2 and Group B2, and between Group A3 and Group B3 (all *P* > 0.05).

**Table 1:** Comparison of preoperative patient parameters (Mean ± SD)

Parameter	Group A1 (*r*<0.3 mm) (27 eyes)	Group B1 (*r* ≥ 0.3 mm) (52 eyes)	*P*	Group A2 (*r* < 0.4 mm) (41 eyes)	Group B2 (*r* ≥ 0.4 mm) (38 eyes)	*P*	Group A3 (*r* < 0.5 mm) (60 eyes)	Group B3 (*r* ≥ 0.5 mm) (19 eyes)	*P*
Age (y)^a^	57.89±11.21	56.90±8.41	0.662	57.51±10.34	56.95±8.40	0.792	55.82±12.27	56.79±10.45	0.756
UDVA (logMAR)^b^	0.96±0.58	0.80±0.44	0.302	0.96±0.54	0.74±0.41	0.088	0.88±0.53	0.76±0.36	0.604
CDVA (logMAR) ^b^	0.57±0.34	0.52±0.30	0.490	0.55±0.33	0.52±0.29	0.819	0.52±0.33	0.58±0.26	0.212
AL (mm) ^b^	23.95±1.01	23.65±1.12	0.142	23.92±0.99	23.58±1.17	0.089	23.88±1.08	23.35±1.03	0.067
ACD (mm) ^b^	3.29±0.44	3.37±0.37	0.602	3.37±0.42	3.31±0.36	0.418	3.36±0.41	3.29±0.33	0.524
Km (D) ^a^	43.78±1.32	43.65±1.73	0.720	43.65±1.39	43.74±1.80	0.809	43.72±1.40	43.60±2.14	0.813
LT (mm) ^a^	4.13±0.49	4.07±0.41	0.563	4.12±0.49	4.06±0.38	0.532	4.11±0.42	4.03±0.48	0.454
Corneal astigmatism (D) ^b^	0.67±0.27	0.62±0.30	0.471	0.65±0.27	0.62±0.32	0.617	0.63±0.28	0.67±0.36	0.590
Mesopic pupil size (mm) ^b^	4.39±0.60	4.24±0.45	0.344	4.36±0.54	4.22±0.47	0.237	4.32±0.51	4.21±0.52	0.226
IOL power (D) ^b^	21.06±2.03	21.61±2.72	0.064	21.24±2.09	21.61±2.90	0.178	21.10±2.65	22.42±1.69	0.058
Target refraction (D) ^a^	-0.55±0.18	-0.56±0.15	0.816	-0.56±0.17	-0.54±0.15	0.639	-0.56±0.16	-0.52±0.15	0.388

*SD* Standard deviation, *UDVA* Uncorrected distance visual acuity, *CDVA* Corrected distance visual acuity, *logMAR* Logarithm of the minimum angle of resolution, *AL* Axial length, *ACD* Anterior chamber depth, *Km* Mean keratometry, *LT* Lens thickness, *IOL* Intraocular lens

^a^ parameters normally distributed

^b^ parameters not normally distributed

### IOL decentration, corneal astigmatism, and refractive outcomes

Table 2 shows the mean postoperative IOL decentration, corneal astigmatism, and refractive outcomes (sphere, cylinder, and MRSE) at 6 months. There were no statistically significant differences in IOL decentration, corneal astigmatism, sphere, cylinder, and MRSE between Group A1 and Group B1, between Group A2 and Group B2, and between Group A3 and Group B3 (all *P* > 0.05).

**Table 2:** Comparison of postoperative IOL decentration, corneal astigmatism, and refractive outcomes (Sphere, Cylinder, and MRSE) (Mean±SD)

Parameter^b^	Group A1 (*r*<0.3 mm) (27 eyes)	Group B1 (*r* ≥ 0.3 mm) (52 eyes)	*P*	Group A2 (*r*<0.4 mm) (41 eyes)	Group B2 (*r* ≥ 0.4 mm) (38 eyes)	*P*	Group A3 (*r*<0.5 mm) (60 eyes)	Group B3 (*r* ≥ 0.5 mm) (19 eyes)	*P*
IOL decentration^a^	0.21±0.10	0.25±0.13	0.222	0.21±0.10	0.26±0.14	0.100	0.23±0.13	0.26±0.08	0.257
Corneal astigmatism (D) ^b^	0.52±0.20	0.52±0.23	0.925	0.52±0.20	0.52±0.24	0.945	0.51±0.21	0.56±0.25	0.304
Sphere (D) ^b^	-0.41±0.39	-0.41±0.31	0.626	-0.37±0.34	-0.46±0.33	0.229	-0.41±0.34	-0.41±0.33	0.868
Cylinder (D) ^b^	-0.45±0.28	-0.48±0.27	0.734	-0.50±0.27	-0.44±0.26	0.304	-0.48±0.27	-0.45±0.27	0.658
MRSE (D) ^b^	-0.63±0.42	-0.65±0.30	0.708	-0.62±0.35	-0.68±0.34	0.540	-0.65±0.34	-0.63±0.36	0.777

*IOL* Intraocular lens, *MRSE* Manifest refraction spherical equivalent

^a^parameters normally distributed

^b^ parameters not normally distributed

### Angle alpha

**Fig. 2:**
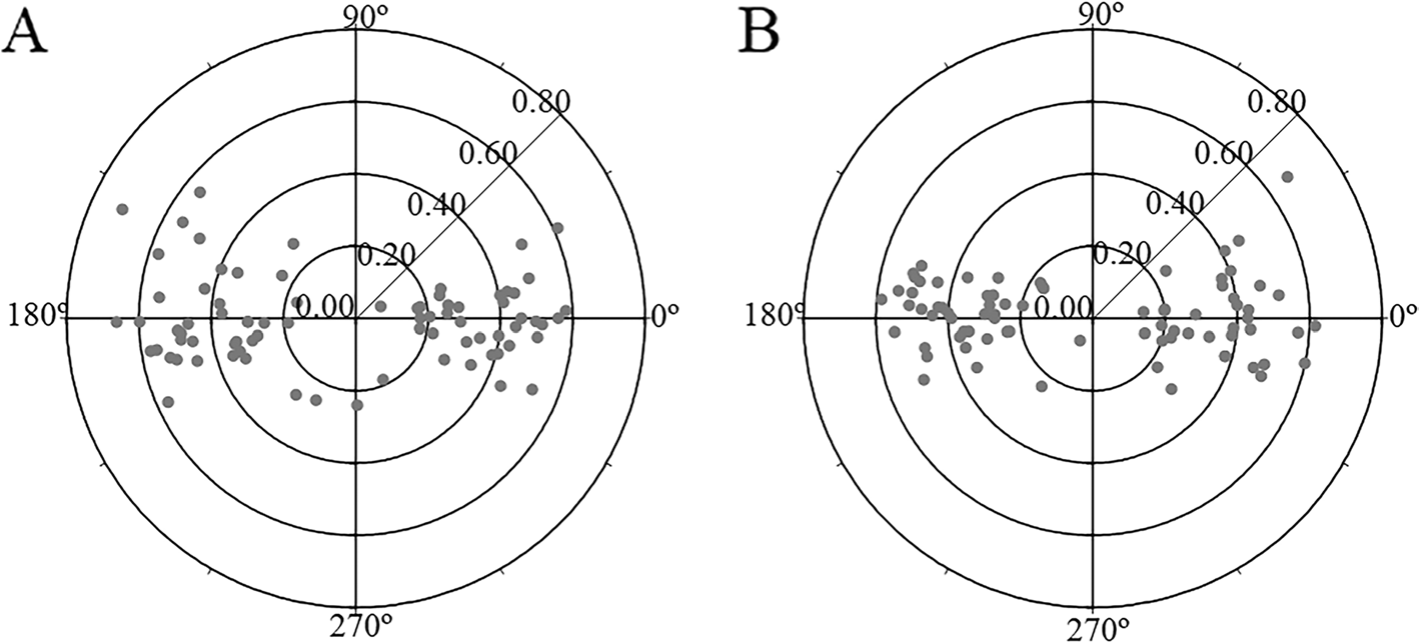
The polar plot graph of preoperative and postoperative angle alpha. **A** The polar plot graph of preoperative angle alpha. **B** The polar plot graph of postoperative angle alpha

Figure 2 shows the polar plot graph of preoperative angle alpha (Fig. 2A) and postoperative angle alpha (Fig. 2B). The mean preoperative angle alpha was 0.39 ± 0.14 mm (range: 0.08 to 0.71). The mean postoperative angle alpha was 0.37 ± 0.13 mm (range: 0.07 to 0.67). There were no statistically significant differences between preoperative and postoperative angle alpha (*P* = 0.314).

**Fig. 3:**
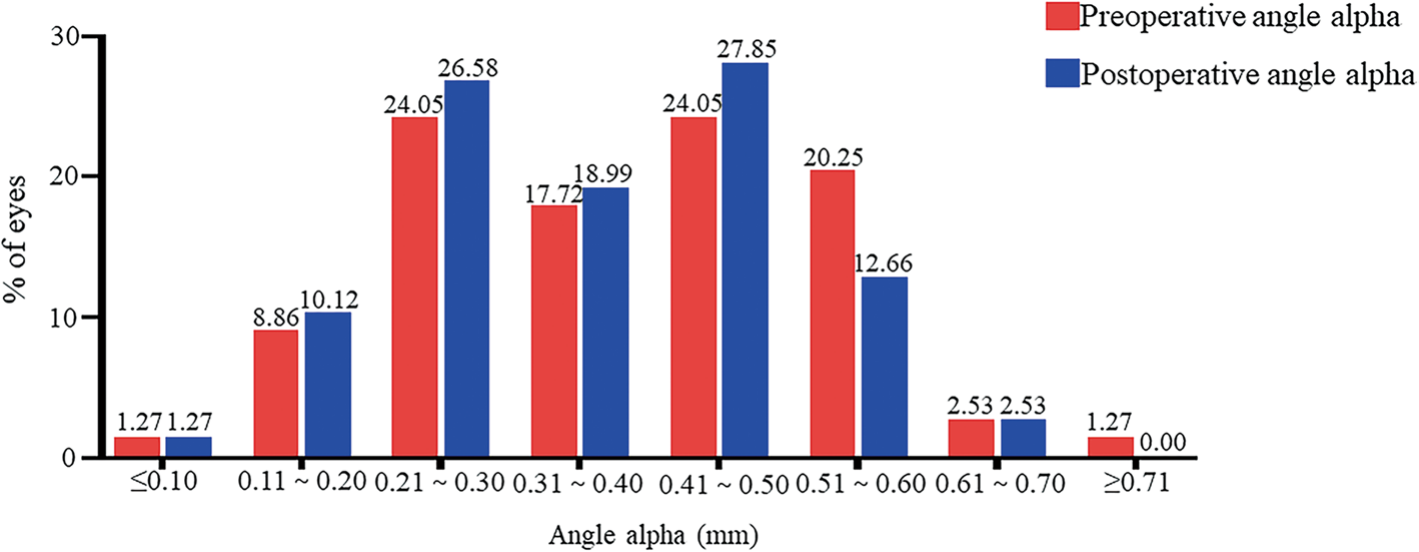
The distribution of preoperative and postoperative angle alpha

Figure 3 shows the distribution of preoperative and postoperative angle alpha. Preoperatively, 27 eyes (34.18%) had an angle alpha <0.3 mm whereas postoperatively 30 eyes (37.97%) had an angle alpha <0.3 mm; Preoperatively, 41 eyes (51.90%) had an angle alpha <0.4 mm whereas postoperatively 45 eyes (56.96%) had an angle alpha <0.4 mm; and preoperatively, 60 eyes (75.95%) had an angle alpha <0.5 mm whereas postoperatively 67 eyes (84.81%) had an angle alpha <0.5 mm. Out of the 79 eyes, postoperatively, 44 eyes (55.69%) decreased after surgery, and 34 eyes (43.04%) increased after surgery, while 1 eye (1.27%) kept changeless after surgery.

### Visual acuity

Table 3 shows the mean postoperative visual acuity results at 6 months. There were no statistically significant differences in UDVA, UIVA, UNVA, CDVA, CIVA, and CNVA between Group A1 and Group B1, between Group A2 and Group B2, and between Group A3 and Group B3 (all *P* > 0.05).

**Table 3:** Comparison of postoperative visual acuity (logMAR) (Mean±SD)

Parameter^a^	Group A1 (*r*<0.3 mm) (27 eyes)	Group B1 (*r* ≥ 0.3 mm) (52 eyes)	*P*	Group A2 (*r*<0.4 mm) (41 eyes)	Group B2 (*r* ≥ 0.4 mm) (38 eyes)	*P*	Group A3 (*r*<0.5 mm) (60 eyes)	Group B3 (*r* ≥ 0.5 mm) (19 eyes)	*P*
UDVA	0.04±0.06	0.06±0.07	0.243	0.05±0.06	0.06±0.07	0.976	0.05±0.06	0.06±0.07	0.943
UIVA	-0.01±0.06	0.00±0.06	0.465	-0.00±0.06	-0.00±0.07	0.712	-0.00±0.06	0.00±0.07	0.787
UNVA	0.13±0.07	0.14±0.07	0.446	0.13±0.07	0.13±0.08	0.895	0.13±0.07	0.14±0.07	0.618
CDVA	-0.02±0.05	-0.00±0.04	0.073	-0.02±0.05	0.00±0.04	0.060	-0.01±0.05	-0.00±0.03	0.493
CIVA	-0.05±0.04	-0.04±0.05	0.273	-0.04±0.05	-0.04±0.05	0.500	-0.04±0.04	-0.04±0.06	0.932
CNVA	-0.01±0.06	-0.01±0.14	0.277	0.01±0.06	-0.03±0.15	0.228	0.00±0.06	-0.06±0.21	0.240

*SD* Standard deviation, *UDVA* Uncorrected distance visual acuity, *UIVA* Uncorrected intermediate visual acuities, *UNVA* Uncorrected near visual acuities, *CDVA* Corrected distance visual acuity, *CIVA* Corrected intermediate visual acuities, *CNVA* Corrected near visual acuities, *logMAR* Logarithm of the minimum angle of resolution

^a^all parameters not normally distributed

### MTF

Table 4 shows the mean postoperative MTF at 6 months with 3 mm and 5 mm pupil diameter. There were no statistically significant differences in 5, 10, 15, 20, 25, and 30 cycles/deg between Group A1 and Group B1, between Group A2 and Group B2, and between Group A3 and Group B3 in 3 mm pupil diameter, so was in 5 mm pupil diameter (all *P* > 0.05).

**Table 4:** Comparison of difference spatial frequencies MTF for 3 mm pupil diameter and 5 mm pupil diameter (Mean ± SD, cycles/deg)

Parameter	Group A1 (*r*<0.3 mm) (27 eyes)	Group B1 (*r* ≥ 0.3 mm) (52 eyes)	*P*	Group A2 (*r*<0.4 mm) (41 eyes)	Group B2 (*r* ≥ 0.4 mm) (38 eyes)	*P*	Group A3 (*r*<0.5 mm) (60 eyes)	Group B3 (*r* ≥ 0.5 mm) (19 eyes)	*P*
3 mm pupil diameter									
5^a^	0.48±0.16	0.45±0.12	0.355	0.46±0.15	0.45±0.12	0.735	0.46±0.14	0.44±0.10	0.572
10^b^	0.20±0.08	0.18±0.12	0.055	0.18±0.07	0.19±0.13	0.453	0.20±0.11	0.16±0.08	0.082
15^b^	0.14±0.06	0.12±0.08	0.133	0.12±0.06	0.13±0.09	0.757	0.13±0.08	0.11±0.06	0.329
20^b^	0.10±0.04	0.09±0.07	0.057	0.09±0.04	0.10±0.08	0.380	0.10±0.06	0.08±0.04	0.068
25^b^	0.08±0.03	0.07±0.04	0.089	0.07±0.03	0.07±0.04	0.610	0.07±0.04	0.06±0.04	0.133
30^b^	0.06±0.02	0.06±0.03	0.109	0.06±0.02	0.06±0.03	0.444	0.06±0.03	0.05±0.02	0.137
5 mm pupil diameter									
5^a^	0.35±0.06	0.32±0.08	0.056	0.34±0.08	0.32±0.06	0.255	0.33±0.08	0.32±0.06	0.667
10^b^	0.14±0.07	0.13±0.06	0.209	0.14±0.07	0.12±0.06	0.280	0.13±0.07	0.13±0.06	0.709
15^b^	0.10±0.07	0.08±0.04	0.100	0.09±0.06	0.08±0.04	0.486	0.09±0.05	0.09±0.04	0.868
20^b^	0.08±0.04	0.06±0.04	0.091	0.07±0.04	0.06±0.04	0.303	0.07±0.04	0.07±0.04	0.692
25^b^	0.06±0.04	0.05±0.03	0.071	0.06±0.03	0.05±0.03	0.241	0.05±0.03	0.05±0.03	0.868
30^b^	0.05±0.02	0.04±0.02	0.063	0.05±0.02	0.04±0.02	0.239	0.05±0.02	0.04±0.02	0.863

*MTF* Modulation transfer function, *SD* Standard deviation

^a^ parameters normally distributed

^b^ parameters not normally distributed

### Aberrations

Table 5 shows the mean postoperative ocular aberration, internal aberration, and corneal aberration at 6 months with 3 mm and 5 mm pupil diameter. There were no statistically significant differences of ocular aberration, internal aberration, and corneal aberration between Group A1 and Group B1 in 3 mm and 5 mm pupil diameter, respectively (all *P* > 0.05). Moreover, with 3 mm pupil diameter, there were no statistically significant differences of ocular aberration and corneal aberration between Group A2 and Group B2 and between Group A3 and Group B3 (all P > 0.05). However, there were statistically significant differences of HOAs and spherical aberration (SA) in internal aberration between Group A2 and Group B2 in 3 mm pupil diameter (all *P* < 0.05). And there were statistically significant differences of HOAs, SA, and trefoil in internal aberration between Group A3 and Group B3 in 3 mm pupil diameter (all *P* < 0.05). In addition, with 5 mm pupil diameter, there were no statistically significant differences of corneal aberration between Group A2 and Group B2 and between Group A3 and Group B3 (all *P* > 0.05). However, there were statistically significant differences of HOAs and SA in ocular aberration and internal aberration between Group A2 and Group B2 in 5 mm pupil diameter (all *P* < 0.05). And there were statistically significant differences of HOAs, coma, and SA in ocular aberration and internal aberration between Group A3 and Group B3 in 5 mm pupil diameter (all *P* < 0.05).

**Table 5:** Comparison of ocular aberration, internal aberration, and corneal aberration for 3 mm pupil diameter and 5 mm pupil diameter (Mean ± SD, μm)

Parameter	Group A1 (*r*<0.3 mm) (27 eyes)	Group B1 (*r* ≥ 0.3 mm) (52 eyes)	*P*	Group A2 (*r*<0.4 mm) (41 eyes)	Group B2 (*r* ≥ 0.4 mm) (38 eyes)	*P*	Group A3 (*r*<0.5 mm) (60 eyes)	Group B3 (*r* ≥ 0.5 mm) (19 eyes)	*P*
3 mm pupil diameter									
Ocular aberration									
HOAs^a^	0.21±0.09	0.24±0.08	0.152	0.22±0.08	0.24±0.08	0.247	0.22±0.08	0.26±0.09	0.077
Coma^b^	0.10±0.06	0.11±0.06	0.251	0.10±0.06	0.12±0.06	0.231	0.11±0.06	0.12±0.06	0.289
SA^a^	-0.05±0.05	-0.04±0.07	0.913	-0.06±0.05	-0.03±0.08	0.097	-0.05±0.06	-0.02±0.08	0.051
Trefoil^b^	0.13±0.08	0.13±0.07	0.691	0.13±0.08	0.12±0.08	0.559	0.12±0.07	0.14±0.09	0.594
Internal aberration									
HOAs^a^	0.21±0.07	0.24±0.07	0.073	0.21±0.07	0.24±0.08	0.042*	0.22±0.07	0.26±0.08	0.024*
Coma^b^	0.09±0.05	0.12±0.07	0.138	0.10±0.06	0.13±0.07	0.114	0.11±0.07	0.13±0.07	0.176
SA^b^	-0.09±0.05	-0.07±0.08	0.660	-0.09±0.05	-0.05±0.08	0.038*	-0.09±0.06	-0.04±0.08	0.034*
Trefoil^b^	0.10±0.06	0.10±0.06	0.294	0.09±0.06	0.11±0.07	0.351	0.09±0.05	0.13±0.08	0.045*
Corneal aberration									
HOAs^a^	0.09±0.03	0.09±0.04	0.476	0.09±0.04	0.09±0.03	0.559	0.09±0.04	0.09±0.03	0.780
Coma^b^	0.04±0.02	0.04±0.02	0.745	0.04±0.02	0.04±0.02	0.713	0.04±0.02	0.04±0.02	0.739
SA^b^	0.04±0.04	0.02±0.03	0.390	0.03±0.05	0.02±0.02	0.143	0.03±0.04	0.02±0.01	0.283
Trefoil^a^	0.07±0.03	0.07±0.04	0.394	0.07±0.04	0.07±0.03	0.535	0.07±0.04	0.07±0.03	0.532
5 mm pupil diameter									
Ocular aberration									
HOAs^b^	0.59±0.30	0.70±0.29	0.056	0.59±0.28	0.73±0.31	0.026*	0.61±0.29	0.82±0.28	0.005*
Coma^b^	0.30±0.11	0.33±0.22	0.792	0.29±0.13	0.35±0.24	0.356	0.29±0.18	0.39±0.21	0.043*
SA^a^	-0.07±0.09	-0.08±0.13	0.785	-0.05±0.09	-0.10±0.13	0.042*	-0.06±0.12	-0.12±0.10	0.042*
Trefoil^b^	0.36±0.23	0.37±0.18	0.360	0.37±0.22	0.36±0.17	0.536	0.35±0.20	0.39±0.21	0.242
Internal aberration									
HOAs^b^	0.52±0.26	0.60±0.28	0.166	0.50±0.22	0.65±0.30	0.018*	0.52±0.26	0.73±0.27	0.002*
Coma^b^	0.24±0.15	0.27±0.18	0.388	0.22±0.13	0.30±0.19	0.084	0.24±0.16	0.33±0.17	0.010*
SA^a^	-0.19±0.08	-0.21±0.12	0.379	-0.18±0.08	-0.23±0.13	0.041*	-0.19±0.10	-0.25±0.10	0.035*
Trefoil^b^	0.25±0.18	0.20±0.12	0.235	0.22±0.16	0.21±0.13	0.891	0.21±0.15	0.26±0.14	0.062
Corneal aberration									
HOAs^a^	0.32±0.11	0.37±0.13	0.104	0.34±0.13	0.37±0.11	0.386	0.35±0.13	0.37±0.12	0.628
Coma^a^	0.13±0.07	0.14±0.08	0.567	0.13±0.08	0.15±0.08	0.336	0.13±0.08	0.15±0.09	0.489
SA^b^	0.12±0.05	0.13±0.04	0.306	0.13±0.05	0.13±0.04	0.624	0.13±0.05	0.13±0.04	0.671
Trefoil^a^	0.23±0.11	0.26±0.14	0.293	0.25±0.13	0.25±0.13	0.777	0.25±0.13	0.24±0.14	0.736

*SD* Standard deviation, *HOAs* Higher-order aberrations, SA Spherical aberration

^a^ parameters normally distributed

^b^ parameters not normally distributed

*Statistically significant

### Correlation between angle alpha and visual quality

The correlation coefficient (r) was obtained between preoperative angle alpha distance and different visual quality parameters (UDVA, UIVA, UNVA, CDVA, CIVA, CNVA, MTF, and ocular aberration, internal aberration, and corneal aberration). The SA in internal aberration in 3 mm pupil diameter (r = 0.222, *P* = 0.049), HOAs in ocular aberration (r = 0.297, *P* = 0.008) and HOAs in internal aberration (r = 0.269, *P* = 0.016) in 5 mm pupil diameter had a statistically significant correlation to preoperative angle alpha. However, none of other parameters had a statistically significant correlation to preoperative angle alpha (all *P* > 0.05).

Table 6 shows the regression analysis results. The regression coefficient indicated that angle alpha had the impact on the SA in internal aberration in 3 mm pupil diameter, HOAs in ocular aberration and HOAs in internal aberration in 5 mm pupil diameter. However, none of other parameters (visual acuity, MTF, and any other aberrations) had a statistically significant correlation to preoperative angle alpha (all *P* > 0.05).

**Table 6:** Regression analysis results between angle alpha and the parameters of visual quality.

Parameter	*R*^2^	*P*	Equation
3 mm pupil diameter			
SA in internal aberration	0.087	0.008	Y = 0.147 * X – 0.131
5 mm pupil diameter			
HOA in ocular aberration	0.079	0.012	Y = 0.607 * X + 0.427
HOA in internal aberration	0.072	0.017	Y = 0.533 * X + 0.364

*SA* Spherical aberration, *HOAs* Higher-order aberrations

### Photic phenomena

**Fig. 4:**
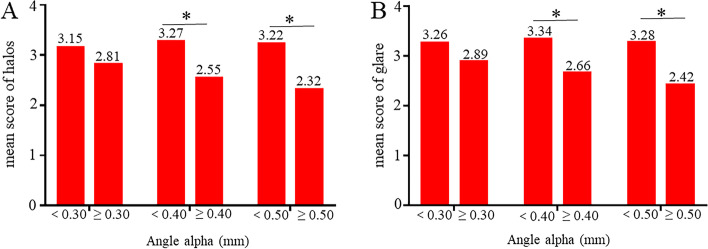
The result of halos and glare. **A** The result of halos. **B** The result of glare

Figure 4 shows mean score of halos (Fig. 4A) and glare (Fig. 4B) reported in the patient questionnaire. The mean scores of halos in Group A1 and Group B1 were 3.15 and 2.81, mean scores of glares in Group A1 and Group B1 were 3.26 and 2.89. The mean scores of halos in Group A2 and Group B2 were 3.27 and 2.55, mean scores of glares in Group A2 and Group B2 were 3.34 and 2.66. The mean scores of halos in Group A3 and Group B3 were 3.22 and 2.32, mean scores of glares in Group A3 and Group B3 were 3.28 and 2.42. There were no statistically significant differences in halos and glare between Group A1 and Group B1 (*P* = 0.259 and 0.268). However, there were statistically significant differences in halos and glare between Group A2 and Group B2 (*P* = 0.008 and 0.013), so was between Group A3 and Group B3 (*P* = 0.005 and 0.007).

## Discussion

EDOF IOLs not only offer excellent distance, intermediate, and near visual acuity, but also provide a better objective visual quality compared to monofocal IOLs [15–17]. However, photic phenomena were one of the main complaints in patients with the implantation of EDOF IOLs after cataract surgery, and were related with angle kappa [1, 3]. Furthermore, due to the indeterminacy of the optical axis, angle alpha is rarely applied in clinic work. With the development of high-precision instruments, iTrace has become the common instrument to measure angle alpha. Previous studies have reported that the causes of undesired postoperative outcomes were dry eye, residual lens fragments, posterior capsule opacification, IOL decentration, astigmatism, and MRSE [18–21]. Our study ruled out the interference of these factors. First, we incorporated dry eye, residual lens fragments, and posterior capsule opacification into exclusion criteria. Second, we ruled out the interference about the age, astigmatism, IOL power and target refraction before cataract surgery. Third, we made comparison of subjective and objective visual quality on the basis of no difference with IOL decentration, astigmatism, and MRSE between groups with different angle alpha after cataract surgery. Finally, a correlation analysis was done between angle alpha and the parameters of visual quality. Therefore, based on the above, we evaluated whether angle alpha changes after implantation of EDOF IOLs, and whether magnitude of angle alpha was associated with a deterioration of postoperative visual acuity, MTF, aberration, and photic phenomena outcomes. A cut-off value of 0.3 mm, 0.4 mm, and 0.5 mm in preoperative angle alpha was chosen to divide eyes into groups to determine which cut-off value is the most suitable index for the implantation of EDOF IOLs.

In our study, we evaluated whether angle alpha changes after the implantation of EDOF IOLs, and found the mean postoperative angle alpha were slightly lower than the mean preoperative angle alpha in all eyes. However, there were no statistically significant differences were found between magnitude of preoperative and postoperative angle alpha. In our study, angle alpha was defined as being the distance between the visual axis and the center of cornea. Since the center of cornea is relatively constant, the change of angle alpha might be related to the change of visual axis after the operation, especially in uneven opacity of the crystalline lens, which may cause the line of sight to change, thus resulting in the change of visual axis after the surgery [10]. Besides, previous studies have indicated that the magnitude of angle kappa after the phacoemulsification significantly decreased due to the change of visual axis and the change of diameter, shape, and position of the pupil [9, 10]. This indicated that angle kappa was relatively unstable respect to angle alpha. According to the above, angle alpha may be a more reliable reference compared to angle kappa for the implantation of MIOLs, which was in accordance with a recently published study [10]. Therefore, angle alpha should be more considered in the preoperative assessment of patients implanted MIOLs in clinic work.

In the present study, the mean postoperative logMAR visual acuity in all groups ranged from 0.30 to − 0.08. This confirmed that the EDOF IOLs can provide good postoperative far, intermediate, and near vision, in term of visual acuity outcomes. Besides, there were no statistically significant differences in far, intermediate, and near visual acuity between all groups, which showed that magnitude of angle alpha did not negatively impact visual outcomes and had no any influence on postoperative vision. This is consistent with recently published article [1].

The MTF is the ratio of contrast between the retinal image and the original scene [1, 20], and this reflects the transmission ability of the optical system to different spatial frequencies. We used 3 mm and 5 mm pupil diameters to simulate the visual quality under photopic and scotopic conditions, respectively. In our study, there were no statistically significant differences in difference spatial frequencies MTF for between all groups with 3 mm and 5 mm diameter, respectively. This showed that MTF was not affected by the magnitude of angle alpha. In a word, the magnitude of angle alpha had no effect on visual image quality of the human eye optical system whether it is under photopic and scotopic conditions.

Aberrations lead to defects in image-forming. This caused the image obtained to being imperfect, thus decreasing the visual quality. Previous studies reported that HOAs near the center of the Zernike polynomials, such as coma, SA, and trefoil, tend to more significantly affect visual quality than those at the periphery of the Zernike polynomials [22, 23]. Therefore, in our study, HOAs mainly included coma, SA, and trefoil. Previous study has shown that HOAs, coma, and SA in ocular aberration and internal aberration were the smallest in the monofocal IOL than those in the MIOL (EDOF IOL and ZMB00 IOL) with 3 mm pupil diameter, and ocular aberrations and internal aberrations were the largest in the ZMB00 IOL than those monofocal IOL and EDOF IOL [24]. Our study found that there were no statistically significant differences of ocular aberration, internal aberration, and corneal aberration between Group A1 and Group B1 in 3 mm and 5 mm pupil diameter, respectively. This showed the cut-off value of 0.3 mm in angle alpha had no influence on aberrations. However, when 0.4 mm in angle alpha was chosen the cut-off value in 3 mm pupil diameter, this caused significant differences of HOAs and SA in internal aberration, whereas there were no significant differences in ocular aberration. The possible reasons were as follows: first, internal aberration represented all aberrations behind the anterior corneal surface. When the implantation of IOL, it mainly caused changes in internal aberration. Second, ocular aberration is composed of internal aberration and corneal aberration, and ocular aberration plays a pivotal role in the visual quality of patients. Under the premise that the corneal aberration was almost constant, the change in internal aberration was not enough to cause a significant change in ocular aberration. This showed the cut-off value of 0.4 mm in angle alpha did not affect ocular aberration in the 3 mm pupil diameter. In a word, the cut-off value of 0.4 mm in angle alpha did not affect the patient’s visual quality under photopic conditions. And so was the cut-off value of 0.5 mm in angle alpha in a 3 mm pupil diameter. Therefore, magnitude of angle alpha did not deteriorate visual quality under photopic conditions. More importantly, when 0.4 mm in angle alpha was chosen the cut-off value in a 5 mm pupil diameter, in addition to HOAs and SA in internal aberration, there also were statistically significant differences of HOAs and SA in ocular aberration, thus leading to the decline of visual quality. Moreover, compared to the cut-off value of 0.4 mm, when 0.5 mm in angle alpha was chosen the cut-off value in 5 mm pupil diameter, in addition to HOAs and SA, significant differences of coma in ocular aberration and internal aberration were also added. It was well known that ocular aberrations increase together with pupil diameter under dim light conditions [25], thus worsening the visual quality of patients. Based on the above, our outcomes indicated that the cut-off value of 0.4 mm or higher in angle alpha mainly affected the visual quality under scotopic conditions. However, magnitude of angle alpha did not affect the visual quality under photopic conditions. To the best of our knowledge, there were few studies evaluating the influence of angle alpha on visual quality, and only one recently published article showed that was no correlation with angle alpha after EDOF IOLs implantation [1], this is inconsistent with our study. The possible reasons were as follows: first, the parameters of objective visual quality were different. Recently published article showed that objective visual quality included objective scattering index (OSI), MTF cutoff, strehl ratio, and simulated visual acuity at 100, 20, and 9% contrast. However, in our study, objective visual quality mainly included MTF, ocular aberration, internal aberration, and corneal aberration. Second, the statistical method between angle alpha and visual quality was not the same. Recently published article calculated their correlation of those visual quality results with angle alpha while our study grouped according to the size of the angle alpha and compared the differences in visual quality between different groups. Therefore, our results might provide a reference for the critical size of the angle alpha necessary for successful implantation of MIOL.

Last but not least, photic phenomena, such as halos and glare, was still the common complaints of patients after the implantation of MIOLs, which seriously affected patient satisfaction [1–3]. In our study, the statistically significant differences in the score of photic phenomena were found in between Group A2 and Group B2 and between Group A3 and Group B3. This showed the cut-off value of 0.4 mm or higher in angle alpha experienced more risk in halos and glare. Previous studies showed photic phenomena were related to be preoperative large angle kappa [26, 27]. Especially when preoperative angle kappa was greater than 0.4 mm, the incidence of glare and halo increased [2]. However, some patients with preoperative large angle kappa had no photic phenomena postoperatively [28]. One possible reason might be that significant reduction in angle kappa was found after cataract surgery compared with pre-operation [10]. Hence, the light enters the eye through the central area of the IOL instead of through other diffraction rings after post-operation, thus not causing photic phenomena. This showed that the magnitude of postoperative angle kappa played a key role in photic phenomena. However, angle alpha, which was similar to angle kappa, played an important role in the implantation of MIOLs [1], and our study showed that angle alpha almost unchanged before and after surgery (pre-operation vs post-operation: 0.39 mm and 0.37 mm), which is in accordance with recent studies [9, 10]. This indicated angle alpha may a more accurate indicator in the preoperative evaluation of cataract patients compared to angle kappa in clinical work. Larger angle alpha indicated measurements are taken further from the optical axis, which caused more photic phenomena. For patients with a 0.4 mm or higher of angle alpha, the choice to implant the MIOLs should be carefully evaluated.

However, this study has several limitations. There was a relatively small number of patients in the cut-off value of 0.3 mm, 0.4 mm, and 0.5 mm in angle alpha, especially the number in between r < 0.3 mm and r ≥ 0.30 mm and between r < 0.5 mm and r ≥ 0.50 mm was relatively unequal, further study with a larger sample size is necessary.

## Conclusions

This study reveals that angle alpha did not affect the visual acuity, but the value of 0.4 mm or higher in angle alpha affected the visual quality under scotopic conditions and occurrence of photic phenomena for the implantation of EDOF IOLs. In clinical work, for patients with 0.4 mm or higher in angle alpha, the choice to implant a EDOF IOL should be carefully considered.

## Supplementary Information

**Additional file 1.**

## Data Availability

The datasets used during the current study are available in the supplementary material file and also obtained from the corresponding author on reasonable request.

## Abbreviations

IOLs: Intraocular lenses
MIOLs: Multifocal intraocular lenses;
HOAs: Higher-order aberrations
EDOF: Extended depth of focus
UDVA: Uncorrected distance visual acuity
CDVA: Corrected distance visual acuity
UIVA: Uncorrected intermediate visual acuities
CIVA: Corrected intermediate visual acuities
UNVA: Uncorrected near visual acuities
CNVA: Corrected near visual acuities
MRSE: Manifest refraction spherical equivalent
MTF: Modulation transfer function
logMAR: Logarithm of the minimum angle of resolution
SA: Spherical aberration
OSI: Objective scattering index
